# Cord Malaria Infection, Complement Activation, Oxidative Stress, Gestational Age, and Birth Weight, Characterized by High *Plasmodium falciparum* Prevalence in Bamenda, Cameroon

**DOI:** 10.1155/2020/7209542

**Published:** 2020-08-11

**Authors:** Oumar Mahamat, Kidio Gisele Ndum, Sumo Laurentine, Ntonifor Ngum Helen

**Affiliations:** Laboratory of Biological Sciences, Faculty of Science, The University of Bamenda, Bamenda, Cameroon

## Abstract

**Background:**

It is unknown whether the presence of *Plasmodium falciparum* malaria parasites in umbilical cord blood denotes activation of complement and oxidative stress to affect the duration of pregnancy and birth weight.

**Methods:**

In a cross-sectional study conducted from January to April 2019 in Bamenda, Cameroon, cord blood samples were collected from 300 women at delivery. Parasitaemia was determined microscopically. Babies' weight and age of gestation were recorded. Plasma levels of complement and oxidative stress were measured by specific tests.

**Results:**

Cord blood malaria prevalence was 21.33%. Babies with an infected cord showed a low birth weight and gestation age than those with uninfected cords. More babies with infected cords had LBW (6.25%) compared to the counterparts (5.50%). The levels of parasitaemia and the babies' weight showed a weak positive correlation. The prevalence of preterm and postterm birth was 4.33% and 24.33% respectively, with a weak negative correlation between the age of gestation and the umbilical cord parasitaemia. There was correlation between cord parasitaemia and levels of complement haemolytic activity titter (CH_50_) and specific classical pathway activity (CPA) in cord blood. CH_50_ and CPA levels, however, were significantly higher in infected cord blood samples, compared with uninfected cord blood samples. CH_50_ showed a negative correlation with the birth weight and gestational age in infected cord blood samples. The levels of total oxidative stress (TOS) and total antioxidant defense were significantly lower in infected cord blood than uninfected. TOS displayed a positive correlation with the density of parasitaemia and a weak negative correlation with the birth weight and gestational age in infected cord blood.

**Conclusion:**

Cord blood infection lowers the complement haemolytic titter, oxygen radicals and total antioxidant defense in neonates. This lowering of complement haemolytic titter and oxygen radical compounds in umbilical cord malaria are associated with low birth weight and preterm birth.

## 1. Introduction

Malaria is a disease caused by parasites belonging to the *Plasmodium* genus. The parasites are transmitted to humans through the bite of infected female Anopheles mosquitoes. Malaria remains the first endemic parasitic disease in the world. In 2017, it was responsible for about 219 million clinical cases of sickness and 435,000 deaths worldwide. About 92% of cases and 93% deaths were reported in sub-Saharan Africa (SSA) [1]. In Cameroon, in 2013 for example, 28.7% of the population in health facilities consulted for malaria, and this scourge was responsible for 22% deaths in general population and 29 to 28% in neonates [2, 3].

Mother-to-child transmission constitutes in malaria as one of the major problems compromising the foetal development. It represents a risk factor for preterm birth, foetal growth restriction, and low birth weight [4–6]. Low birth weight that results constitutes a cause of foetal and neonatal morbidity and one of the important determinants of infant healthy growth and development [7, 8]. Mother malaria-related low birth weight is estimated to up to 20% in an endemic area, with a relatively high risk of preterm delivery [4, 9].

However, the mechanism that leads to low birth weight or preterm delivery in vertical transmission of malaria was not clearly understood. Diverse factors are suggested to be responsible. Anaemia related to immune activities are thought to be associated with malaria-related low birth weight [5, 6, 10]. The complement system, an essential part of innate immunity, may play a role. It is known to be important in initiation of acquired immune responses in malaria-infected individuals [11]. During malaria infection, the level of C5a has been described to be high [11, 12]. The increased level of C5a may induce the release of reactive oxygen species which indirectly contribute to foetal growth restriction.

In the past decades, numerous researches showed a wide-spread involvement of the complement system in a number of placental malaria processes [13]. Thus, measurement of the complement system and oxidative stress levels in cord blood serum may be an important tool to assess the disease impact and the health state of neonates in malaria. Identifying the cause of low birth weight and preterm delivery in malaria has been the focus of many studies, and several mechanisms have been proposed over the past decades. However, there is a lack of consensus concerning validation, standardization, and reproducibility. Here, we aim to assess the prevalence of umbilical cord malaria and discuss the contribution of the complement system through oxidative stress in malaria-related low birth weight and preterm delivery.

## 2. Materials and Methods

### 2.1. Study Area

The study took place at the Regional Hospital of Bamenda. Bamenda is the capital of the Northwest Region, Cameroon. Bamenda lies between latitude 5°94′N and 5°98′N and longitudes 10°15′E and 10°18′E [14]. It sits along the Cameroon Volcanic Line with two distinct relief features: a High Lava Plateau of about 1,400 m, and the Lower Plateau, with an average altitude of 1100 m above sea level both separated by a vast escarpment. It has a tropical climate with two seasons, a long rainy season of eight months (March to October) and a short dry season of four months. Malaria transmission in Northwest Cameroon is perennial but seasonal and peaks during the rainy season. *Plasmodium falciparum* is responsible for more than 90% of malaria infections [2].

### 2.2. Study Population

The study participants were pregnant women who voluntarily consented to permit collection of blood from their cord during labour and satisfied the inclusion criteria. Inclusion criteria were confirmation of the active phase of labour and asymptomatic, nonfebrile parturient and singleton pregnancy without any known congenital anomaly. Exclusion from the study were subjects that refused to participate in the study; presence of any medical illness such as diabetes mellitus, chronic renal disease, haemoglobinopathies, HIV/AIDs, filarial infection, chronic hypertension, and severe pre-eclampsia/eclampsia; and presence of multiple gestation, intrauterine foetal death, and obvious foetal anomalies. There were 300 women recruited having met the inclusion criteria.

### 2.3. Clinical Procedures

Women delivering at the health facility, after giving informed consent, were asked a standard series of questions focused on sociodemographic characteristics, history of fever, antimalarial drug use, clinical malaria during pregnancy, and the use of antimalarial chemoprophylaxis. During labour, five millilitres of blood were drawn by venepuncture from the clamped cord immediately after delivery. Neonates and placentas were weighted with an electronic digital scale (±10 grams). The Dubowitz scoring system was used to estimate the gestational age, using findings from physical and neurologic examinations. Scoring by the APGAR index was performed at delivery but was not recorded in this study. Blood samples were separated into two: 1 ml into ethylenediamine tetra acetic acid (EDTA) tubes and the remaining in tubes without EDTA. Serum was collected from the tubes without EDTA.

### 2.4. Parasitological Analysis

Thick smears of cord blood were prepared from each sample, stained with Giemsa stain, and examined for the presence of malarial parasites using routine microscopy to determine parasitaemia. For positive slides, *t* (F1) [15, 16]. Parasite density was graded as low (parasites < 1,000/*µ*L), moderate (1,000–4,999/*µ*L), and high (>5,000/*µ*L) as reported by Owa et al. [9]. Parasite densities (parasite/*µ*l of whole blood) were then calculated. All slides were double-read. If the ratio of densities from the first two readings was greater than 1.5 or less than 0.67 or if fewer than 30 parasites were counted with a difference of more than 10 parasites between the two readings, the slide was evaluated a third time. The geometric mean of the parasite density of the two most concordant results of the three readings was taken as the final result.

(1) $$\begin{array}{c} \text{Parasites}/µ\text{l blood}=\frac{\text{Number of parasites }\mathrm{cunted}\;x\;8000\;\mathrm{WBC}/µ\text{l}}{\text{Number of WBC counted}}. \end{array}$$

### 2.5. Determination of Haemolytic Complement Titters

Complement titters were based on the classical haemolytic assay (CH50) for lysis of antibody-sensitized sheep E. [17, 18] In detail, 2 × 10^8^ E/ml were opsonized with rabbit anti-sheep haemolysin (Sigma-Aldrich, MO) by adding an equal volume of a 1 : 160 dilution of haemolysin to the E suspension, and the mixture was, then, incubated at 37°C in a shaking water bath for 30 minutes. The sensitized E were, then, centrifuged at 3,000 rpm for five minutes and washed three times with gelatine veronal buffer (GVB) (Sigma-Aldrich Inc, supplemented with 10 mM EDTA (GVBE). Sensitized E were stored in GVBE supplemented with 2.5% glucose, 0.03% NaN_3_ at 4°C for not more than two weeks. For use, sensitized erythrocytes were washed with GVB containing Mg^2+^ and Ca^2+^ (GVB^++^).

Cord blood sera (in duplicates) were, then, serially diluted in a 96 micro-well plate (NUNC) in GVB^++^ to give a final dilution of 1/320. To each serum dilution, 25 *µ*l of 5% sensitized E was added followed by incubation at 37°C for 60 min. The reaction was stopped by adding 100 µl of GVBE to all wells. Control wells included spontaneous lysis of opsonized E without serum and 100% lysis well in which complete lysis was achieved by adding 1% triton X. The plate was centrifuged at 3000 rpm in a plate centrifuge at 4°C for 5 min to pellet unlysed cells. 100 *µ*l of supernatant was, then, transferred to a flat-bottomed plate, and the absorbance readings of released haemoglobin were read at 415 nm using a microplate reader. The degree of lysis (Y) was determined from the formula (2) [18, 19]. The serum titter that causes 50% haemolysis of sensitized sheep E was determined using SPSS software.

(2) $$\begin{array}{c} \text{Y}=\frac{\text{OD sample}-\text{OD spontaneous lysis control}}{\text{OD triton lysis}-\text{OD spontaneous lysis control}}. \end{array}$$

### 2.6. Determination of Functional Complement Activity in the Pathways

The functional complement activity in classical and alternative pathways was evaluated using ELISA kits (Sigma) supplied precoated with specific activators for the classical pathway (CP) and alternative pathway (AP). Serum samples were, then, diluted into buffers containing specific blockers of each pathway. Samples were diluted at 1 : 100 and 1 : 18 for the CP and AP, respectively. Positive and negative controls with known activities were used for quality control of the assay and for calculation of the functional activities.

In detail, 100 *µ*l of each diluted sample and control were added in duplicate to the wells of the respective pathway-specific plates and incubated for 70 min at 37°C. Wells were, then, aspirated and washed thrice with wash buffer. Then, 100 *µ*l of conjugate containing alkaline phosphatase-labelled antibodies to C5b-9 was added to each well and incubated for 30 min at 25°C. Wells were again washed thrice, and 100 *µ*l of substrate ((3,3′,5,5′-tetramethylbenzidine (TMB)) was added, and the samples were incubated for 30 min at 25°C. Absorbance was, then, immediately read at 405 nm. Functional activity for the respective pathways was, then, calculated using the OD values of the positive and negative controls.

### 2.7. Estimation of Plasma Total Oxidative Stress

Total oxidative stress (TOS) was measured by quantifying alkoxyl (RO∗) and peroxyl (ROO∗) radicals as described by Piyashi et al. [19] One hundred microliter (100 *µ*l) of plasma diluted to 20 times in PBS was dissolved in 1 ml of acetate buffer. 25 *µ*l of working chromogen (N, N-dimethyl-*p*-phenylenediamine sulphate) solution was, then, added, and absorbance was taken at 505 nm by 6 minutes time-scan in a UV-VIS spectrophotometer. The absorbance values were obtained at 4 to 6 minutes for each sample against blank. The quantity of radical compounds was determined as proportional to the absorbance.

### 2.8. Estimation of Plasma Total Antioxidant Defense

Total antioxidant defense (TAD) was determined as reported by Piyashi et al. [19] based on the measurement of the reduction of the radical cation of the chromogen (N,N-dimethyl-*p*-phenylenediamine sulphate) by antioxidant compounds in the sample. In detail, 1 ml of acetate buffer (pH = 5.2) taken in a test tube, 25 *µ*l chromogen reagent that contains N,N-dimethyl-*p*-phenylenediamine sulphate, and 10 microliter FeCl_3_ solutions are added. Thereafter, 10 *µ*l of 20 times diluted plasma was added, and the absorbance was photometrically read at 505 nm at 37°C. Total antioxidant defense of the sample was considered to be inversely correlated to the absorbance.

### 2.9. Definitions

Blood film results were considered to be positive if any asexual-stage parasites were identified and negative if no parasites were seen in 100 high-power fields. Prematurity was defined as a gestational age <37 weeks (≤245 days), normal gestation age 251–280 days, and postterm >280 days as estimated by Dubowitz examination [9, 20] and low birth weight as a birth weight of <2500 g, normal body weight 2.5–4 kg, and macrosomia (>4 kg) with a gestational age ≥37 weeks [9].

### 2.10. Statistical Analysis

Data collected in the study questionnaire were verified and, then, double-entered. Data were analyzed using Graph Pad Prism software v. 5.20 and SPSS. The analysis included data from births of all enrolled participants. Continuous normally distributed data were described by their means and standard deviation. Proportions were compared using the Chi-square test, and normally distributed continuous variables were compared using the Student *t* -test. Statistical results were considered significant when the two-sided *P* value was ≤0.05.

### 2.11. Ethical Considerations

The study was discussed with health authorities and local leaders to obtain their assent. The study was reviewed and approved by the Faculty of Health Science ethical committee, University of Bamenda. Informed consent was obtained after the consent document was read to the women in the local language. For illiterate mothers, the informed consent discussion process was witnessed by an impartial individual.

## 3. Results

### 3.1. Characteristics of Recruited Women

Data were collected from three hundred women, with a majority (88%) aged between 20 and 35 years. Amongst these women, 98.33% were taking fansidar, and only 8 (2.66%) had clinical malaria during pregnancy (Table 1).

### 3.2. Labour Outcomes

The mean value of the gestational age of women who participated in this study was 275.27 days (±16.33), with a majority delivering at the normal time with normal babies' birth weight. The mean (SD) placenta weight was 606.66 g (±116.69), with a birth/placenta weight ratio of 5.38 (Table 2).

### 3.3. Umbilical Cord Malaria Infections

Out of the 300 cords, there were 64 (21.33%) positive blood films. The density of parasitaemia varied from low to high density. There were 18 (28.12%), 36 (56.25%), and 10 (15.62%) of the cords that showed low, moderate, and chronic infection, respectively (Table 3).

### 3.4. Impact of Parasitaemia on Labour Outcomes

With respect to infection, birth weight and gestation age were significantly lower in women with umbilical cord malaria infection than in women who did not have placental malaria infection (*P* ≤ 0.05). Relative to the density of parasites, this showed that the gestational age had a positive correlation (*r* = 0.14), while the birth weight showed a negative correlation (*r* = −0.16). However, these relationships were not significant (*P* > 0.05; Table 4).

### 3.5. Relationship between the Umbilical Cord Malaria Infections and Total Complement Haemolytic Activity and Specific Pathways Activation of Umbilical Cord Serum

**Figure 1:**
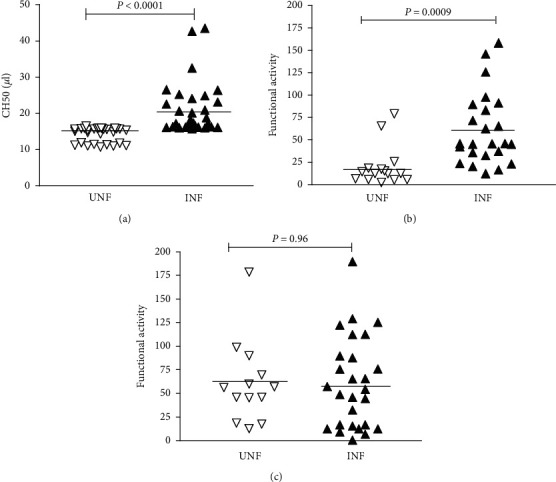
Haemolytic complement titter (CH50) and classical and alternative pathway activity of cord blood serum in relation to malaria parasites in cords. INF (malaria parasite-infected cords) and UNF (malaria parasite-uninfected cords). (a) CH50. (b) Classical pathway. (c) Alternative pathway.

Figure 1 shows the total haemolytic complement titter (CH_50_) and specific complement classical and alternative activities of umbilical cord serum. The Levels of CH_50_ were significantly higher (*P* < 0.0001) in malaria-infected cords (INF), (CH_50_ = 21.41 ± 7.35 UEq/mL) than in noninfected cords (UNF), (CH_50_ = 14.25 ± 2.06 UEq/mL). In specific pathways, only the activities of the classical pathways showed a significantly higher difference at *P* ≤ 0.0001 (% activity = 60.91 ± 40.06%) in INF than in UNF (% activity = 19.91 ± 22.24%). In linear regression, the parasitaemia density showed a significant and negative correlation with total haemolytic complement (*r* = −0.47; *P*=0.005).

### 3.6. Relationship between Labour Outcomes and Complement Haemolytic Activity Titter of Cord Blood Serum with Respect to the Umbilical Cord Parasitaemia

In umbilical cord malaria-positive women, CH_50_ was observed to correlate positively with the gestational age and the baby's birth weight, while in umbilical cord malaria-negative women, it was positively and negatively correlated with the gestational age and the baby's birth weight, respectively. Comparative analysis showed that, in relation to the gestational age, either the delivery was premature, normal, or postterm, and the CH_50_ was high in women with infected cords than in counterpart controls. In contrast, the specific classical pathway activity was low in umbilical cords without parasites than in infected cords for any category of delivery (Table 5).

### 3.7. Impact of Umbilical Cord Malaria Infections on Cord Blood Serum Oxidative Stress and Total Antioxidant Defence

**Figure 2:**
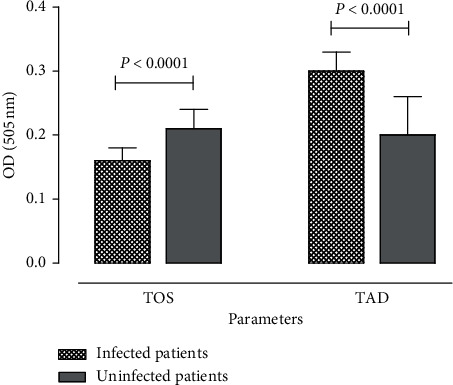
Optical density related to total oxidative stress and total antioxidant defence of cord blood plasma in relation to cord malaria infection. Chart represents Mean ± SD of optical density (*n* = 2).

Determination of the total oxidative stress (TOS) and total antioxidant defence (TAD) was based on the optical density, whose values are known to be positively and negatively correlated with total oxidative stress and total antioxidant defence. Here, in relation with the TOS of the cord blood serum, data showed that the optical density value was significantly (*P* < 0.0001) lower in case of INF compared to the corresponding UNF (healthy control group). When evaluating the TAD, optical density was significantly (*P* < 0.0001) high in case of INF as compared to UNF cords (Figure 2). Parasitaemia density was found to be significantly associated with total oxidative stress (*r* = 0.89, *P* < 0.0001) and total antioxidant defence (*r* = 0.59; *P*=0.0003), with a positive correlation.

### 3.8. Role of Total Oxidative Stress and Total Oxidative Defense in the Labour Outcomes with Respect to the Umbilical Cord Density of Parasitaemia

In case of preterm delivery, as well as in normal and postterm deliveries, compared to UNF cords, the optical density recorded in determination of the total antioxidant defence and total oxidative stress was, respectively, high and low in infected cords. In infected cords, data showed that the gestational age was positively correlated (*r* = 0.11; *P*=0.53) with the TOS, while it was negatively correlated (*r* = −0.88; *P* < 0.0001) with TOS in uninfected cords (Table 6).

Independent on the birth weight value (low, normal, and high), a high optical density characterizing a low serum TAD was observed in uninfected cords compared to infected cords. However, in case of low birth weight, the optical density indicating the serum TOS was high in infected cords. Furthermore, data showed that the birth weights correlated negatively (*r* = −0.15; *P* = 0.41) and positively (*r* = 0.58; *P*=0.0005) with TOS in the umbilical cord with malaria and without malaria, respectively (Table 6).

## 4. Discussion

This study aimed at assessing the prevalence of umbilical cord malaria and discussing the contribution of the complement system through oxidative stress in malaria-related low birth weight and preterm delivery at the regional hospital in Bamenda, Cameroon. The main outcomes of the present study were that the vertical transmission of malaria was 21.33%. This prevalence was despite the intermittent preventive treatment (IPT) based on fansidar. Thus, cord malaria should be given greater attention in malaria control strategies that might include changes of actual drugs, fansidar. Clinical malaria of a mother during pregnancy has been shown not to be associated with cord infection. Thus, asymptomatic malaria of pregnant women should be given greater attention in the prevention of vertical transmission of malaria.

In the current study, the frequency of cords with high density of parasitaemia was higher than the frequency of cords of women with clinical malaria during pregnancy. This demonstrated that vertical transmission of malaria is not a matter of having a clinical malaria in the mother during pregnancy (or peripheral parasitaemia >5000 parasites/*µ*L) as previously reported [21]. Maternal malaria during pregnancy, even in case of low parasitaemia, can result in vertical transmission.

The findings of this study showed a high percentage of preterm delivery and low birth weight in women with cord malaria. Likewise, previous studies demonstrated the impact of the vertical transmission on the baby's body weight at birth and the duration of pregnancy. Preterm birth, foetal growth restriction, and low birth weight were associated with congenital malaria [22–24].

In the present study, the cord blood plasma haemolytic titter of the complement system (CH_50_) was significantly higher in the infected cord than their counterpart control uninfected. The presence of malaria parasite in the cord might activate the complement system, and subsequently, it results in low CH_50_. In a specific pathway functional screen test, data showed that the cord blood plasma of infected cords had high specific classical pathways activities than those of noninfected cords. This suggests that the classical pathway activation, a strict antibody-dependent pathway, might not be affected as the alternative pathway which was low in infected cords. The complement system is a fundamental element of the normal host defence against malaria infection [10]. A generally low value of this immunity component suggests that cord malaria might result in the disruption of immune responses in the neonates.

Furthermore, plasma levels of cord total oxidative stress and total antioxidant defence were significantly lower in malaria-infected than in noninfected women. This finding raises the important question of whether the presence of parasites in cord blood results in oxidative stress in neonates. Oxygen radicals have been demonstrated to be important for the clearance of parasites [25, 26]. Therefore, low oxygen radicals as observed in this study suggest that the high prevalence of umbilical cord malaria may demonstrate inactivity of immune cells, notably phagocytes, the main source of oxygen radicals. Low oxygen radicals of infected cords might also be attributed to low complement components serving in opsonization processes and known to induce the oxidative stress status.

The complement system has been linked to many disease symptoms and complications, including poor pregnancy outcomes [10, 27, 28]. In the current study, serum blood CH_50_ was observed to correlate positively with the gestational age and the baby's birth weight in a malaria-infected cord, while in noninfected cords, it was negatively correlated with the baby's birth weight. Placental malaria was also suggested to be the main determinant of low birth weight [7, 29]. The positive correlation between CH_50_, birth weight, and gestational age, as observed in this study, suggests that the activation of complement resulting in low CH_50_ might be the cause of low birth weight. This is in line with recent observations that showed that soluble terminal complement complex levels were higher in placental plasma samples of newborns weighing less than 2700 g than in heavier newborns [10, 28] as the activation of the complement system that results in low CH_50_ can result in high soluble terminal complement complex levels.

Furthermore, the total oxidative stress was positively correlated with the gestational age, but negatively correlated with the birth weight. Low radical oxygen as observed in malaria-infected cords in consequence of a low complement system subsequent to infection might induce preterm birth or low birth.

## 5. Conclusions

Findings of this study revealed that cord malaria remains prevalent in Bamenda, Cameroon, with a high rate of asymptomatic carriage of malaria parasites. The level of cord infection significantly influenced the birth weight and gestational age. In addition to the cord blood parasitaemia that constituted a source of preterm delivery and low birthweight, the low complement activities, low total oxidative stress, and antioxidant defence of cord blood during vertical transmission might reveal an additional interesting cause of low birth weight and preterm delivery in malaria endemic areas.

## Figures and Tables

**Table 1:** Overall prevalence of malaria with respect to demographic information.

Characteristics	Frequency	Percent (%)
*Age groups (yrs)*		
Age < 20 years	19	6.33
Age between 20 and 35 years	264	88
Age > 35 years	17	5.66
*Chemoprophylaxis*		
No	5	1.66
Yes	295	98.33
*Clinical malaria during pregnancy*		
Yes	8	2.66
No	292	97.33

**Table 2:** Distribution of the studied population according to the gestational age and birth weight.

Characteristics	Frequency	Percent (%)
*Gestation age or days from ovulation (days)*		
Preterm (≤245 days)	13	4.33
NGA (251–280 days)	214	71.33
Postterm (>280 days)	73	24.33
Mean ± SD	275.27 ± 16.33	
*Birth weight (kg)*		
LBW (˂2.5 kg)	17	5.66
NBW (2.5–4 kg)	263	87.66
Macrosomia (>4 kg)	20	6.66
Mean birth weight ± SD	3.21 ± 0.58	
*Placental weight (g)*		
Mean placenta weight ± SD	606.66 ± 116.69	
Bw/Pw ratio	5.38 ± 0.83	

Bw, body weight. Pw, placenta weight. LBW, low birth weight. NBW, normal birth weight. NGA, normal gestation age.

**Table 3:** Prevalence of umbilical cord blood malaria and the level of parasitaemia density/*µ*l in Bamenda Regional Hospital, Cameroon.

	Mean ± SD	Frequency	Percent (%)
*Prevalence of malaria parasitaemia in the cord*			
Not infected	—	236	78.66
Infected	3435.5 ± 4366.72	64	21.33
*Classification of malaria parasitaemia density/µl of cord*			
Low	696.88 ± 188.69	18	28.12
Moderate	2448 ± 1214.08	36	56.25
High	11920 ± 5365.90	10	15.62

Low (parasites < 1000/*µ*L); moderate (parasites between 1000–4999 *µ*L); high (parasites > 5000/*µ*L).

**Table 4:** Labour outcomes in relation to the presence of malaria parasites in umbilical cords.

Labour outcomes	Mean values of factors associated with infection		
	(+) cords	(−) cords	*P* value
Gestation age (days)	274.6753 ± 0.18	279.77 ± 3.21	0.048
Birth weight (kg)	3.188 ± 0.59	4.32 ± 0.50	0.041
Placenta weight (g)	610.38 ± 119.61	592.97 ± 104.98	0.290
^*∗*^Percentage of low birth weight (%)	6.25	5.50	

^*∗*^Number of neonates with low birth weight.

**Table 5:** Complement haemolytic titer and classical pathway activity of cord blood serum following the delivery date and birth weight levels.

Delivery outcomes	Umbilical cord malaria-negative women		Umbilical cord malaria-positive women	
	CH_50_	CPA	CH_50_	CPA
Age of gestation				
Less than 245 days	14.6 ± 2.09^a#^	64.5 ± 32.0^a#^	23.6 ± 1.8^b#^	10.9 ± 2.9^b#^
245 days to 280 days	14.7 ± 1.82^a#^	71.1 ± 31.8^a#^	20.1 ± 4.6^b#^	31.5 ± 26.2^b#^
280 days and above	13.6 ± 2.34^a#^	75.6 ± 37.2^a#^	23.7 ± 10.6^b#^	23.2 ± 21.5^b#^
*r* (*P* value)	0.83 (*P* < 0.0001)		0.14 (0.42)	
Baby's weight at birth				
Less than 2.5 kg	13.1 ± 2.5^a#^	33.9 ± 21.4^a#^	24.4 ± 2.1^b#^	11.5 ± 3.4^b#^
2.50 kg to 4.0 kg	14.6 ± 1.8^a#^	68.4 ± 29.4^a#^	20.2 ± 0.3^b&^	19.4 ± 4.7^b&^
High than 4.0 kg	12.5 ± 2.8^a#^	95.5 ± 48.5^a#^	20.7 ± 1.1^b&^	17.9 ± 0.8^b&^
*r* (*P* value)	−0.65 (*P* < 0.0001)		0.008 (0.96)	

CH_50_ (complement haemolytic titter), CPA (classical pathway activation), *r* (Person's correlation coefficient with CH_50_). Symbol letters indicate the difference following delivery outcomes, while letters show the difference related to the umbilical cord malaria.

**Table 6:** Total oxidative stress and total oxidative defence of cord blood serum following the delivery date and birth weight levels.

	Umbilical cord malaria-negative women		Umbilical cord malaria-positive women	
	TAD	TOS	TAD	TOS
Age of gestation				
Less than 245 days	0.28 ± 0.02^a#^	0.14 ± 0.03^a#^	0.13 ± 0.01^b#^	0.19 ± 0.01^b#^
245 days to 280 days	0.29 ± 0.03^a&^	0.16 ± 0.06^a&^	0.18 ± 0.05^b&^	0.21 ± 0.03^b&^
280 days and above	0.30 ± 0.04^a&^	0.18 ± 0.02^a&^	0.18 ± 0.04^b&^	0.21 ± 0.03^b&^
*r* (*P* value)	−0.84 (<0.0001)		0.11 (0.53)	
Baby weight at birth				
Less than 2.5 kg	0.31 ± 0.03^a#^	0.12 ± 0.02^a#^	0.15 ± 0.03^b#^	0.20 ± 0.01^b#^
2.50 kg to 4.0 kg	0.27 ± 0.03^a&^	0.16 ± 0.11^a&^	0.19 ± 0.05^b&^	0.15 ± 0.03^a&^
High than 4.0 kg	0.26 ± 0.04^a&^	0.18 ± 0.01^a&^	0.18 ± 0.05^b&^	0.16 ± 0.01^b&^
*r* (*P* value)	0.58 (0.0005)		−0.15 (0.41)	

TAD (total oxidative stress), TOS (total oxidative defence), *r* (Person's correlation coefficient with TOS). Symbol letters indicate the difference following delivery outcomes, while letters show the difference related to the umbilical cord malaria.

## Data Availability

All relevant data generated and analyzed during this study are available within the article.
